# Assessment of heavy metals in food and drug packaging materials

**DOI:** 10.12688/f1000research.121473.2

**Published:** 2023-07-07

**Authors:** Senna Mukhi, M. S. Rukmini, Poornima Ajay Manjrekar, Reghupathi Iyyaswami, Sindhu H.

**Affiliations:** 1 Department of Biochemistry, Kasturba Medical College, Mangalore, Manipal Academy of Higher Education, Manipal, India; 2 Department of Chemical Engineering, National Institute of Technology (NITK), Suratkal, Mangalore, India

**Keywords:** Drug, Food, Heavy Metal, ICP-OES, Packaging Material

## Abstract

**Background:** Food and drug packaging materials are an integral part of our everyday life.  Noxious elements can inadvertently be included in packaging materials in various stages of their production. Adulterants, adhesives, colorants and heavy metal interference are the common sources of contamination in food packaging materials. Heavy metal toxicity has far-reaching ill effects on living organisms. The present study aimed at qualitatively and quantitatively analysing heavy metal contamination of various materials that are used for food and drug packaging in India.

**Methods:** The qualitative detection was done by rapid assay and heavy metals were quantified with the help of inductively coupled plasma-optical emission spectrometry (ICP-OES). A total of 13 types of food and drug packaging materials were procured from local market and analysed for four heavy metals *viz.* arsenic (As), vanadium (V), mercury (Hg) and cadmium (Cd). The concentration of each heavy metal in the samples was compared with permitted values published by the European Council.

**Results:** Of the 13 samples, heavy metals were qualitatively detected in 10 samples. ICP­-OES values for quantitative estimation showed presence of heavy metal above permissible range in 10 of the studied samples for vanadium, all samples for arsenic, two samples for mercury and one sample for cadmium. Arsenic was found to be the commonest heavy metal contaminant, present in 13 samples above permissible limit.

**Conclusions:** The significantly higher concentration of heavy metal poses a potential health risk to the consumer and affects the quality of the food.

## Introduction

Packaging materials are defined as any substance or item that comes in contact with food and drug including containers, cans, bottles, cartons, boxes, cases and covering material such as foil, film, metal, paper, wax paper or cloth. ^1^ The main objective of packaging is to provide protection from foreign materials while in-transit and help in maintaining the shelf life of food products. Packaging is a field combining science and technology used for protecting products during distribution, storage, sale and use. It can also be applied to the procedure of evaluating, designing and manufacturing a packed item. Storage and preservation instructions, directions for use, expiry or use by date, and design of packaging materials among others are crucial information which give cognoscibility to the brand and increase the visibility on the shelf. ^2^^,^^3^

All the above processes result in migration of additives, adulterants and toxins such as colourants and adhesives from packaging material into the food, as shown by previous research. ^4^^–^^6^ Leaching can be explained as any process that allows transfer from surface to the core particles. ^7^ With reference to food packaging, the term “leaching” can be defined as the movement of undesirable particles from packaged materials to the packaged particle. The European Council Standard requires that various contaminants like aromatic amines, benzophenones, polyaromatic hydrocarbons, plasticizers and heavy metals be controlled and analyzed in food packages. ^8^ Heavy metals are a huge source of environment pollution. ^9^ The toxicity of heavy metals has harmful effects on biological systems as they do not undergo biodegradation. They accumulate in living organisms, causing several diseases and disorders even when present in very low concentrations. ^10^

The toxic effects of vanadium are particularly seen in lung and stomach tissues, resulting in pneumonia, bronchitis and breach in the gastric mucosal lining. ^11^ Increased concentration of arsenic in humans can induce epigenetic changes and genetic mutations causing cancer. ^12^ Cadmium has been used in industries for a long time. Toxicity due to cadmium can affect kidneys, and the reproductive, skeletal, and respiratory systems, causing proteinuria, kidney stones, loss of bone density and mineralisation, destruction of mucous membranes, pneumonitis, testicular necrosis, and affects steroid hormone synthesis. ^13^ Mercury pollution impacts human health leading to developmental flaws in children and Minamata disease ^14^ Metals like vanadium and mercury may be added as additives as a part of manufacturing process or may be an unintentional adulterant released from the moulds used specifically in the plastic industry. ^15^

In this study locally procured food and drug packaging materials were used. The study started with digestion of packaging materials followed by preliminary qualitative analysis. All the samples were analysed for four heavy metals using spot test for vanadium, senSafe Boris’s mercury detection strips, swab test for cadmium and aquasol arsenic detector kit. After analysing the presence or absence of heavy metals all the samples were quantified using ICP -OES (Inductively coupled plasma optical emission spectrophotometry). The use of ICP-OES is advantageous for sample preparation since it eliminates the need for several dilutions because it can identify multiple components from an analysis. When plasma energy is applied externally to a sample, the constituent elements are stimulated and excited atoms return to their low energy positions leading to emission of rays and the spectrometer captures these to calculate the photon wavelength. The position of the photon rays determines the element type and the strength of the rays establishes the component of each element. ^16^ The detection for arsenic (As), vanadium (V), mercury (Hg) and cadmium (Cd) was quantified in ppm (parts per million). The ICP -OES can detect heavy metals from 0.0002 ppm to 1000 ppm range. ^17^

Considering the wide applications of the packaging material, the concentration of high amounts of heavy metals in them requires fine regulation. The Environmental Defence Fund ^18^ states that these heavy metals can be toxic and the route of ingestion can be packaging materials. ^19^ Sometimes, odorous compounds from the food packaging may get transferred to the food items and affect the foods’ flavour. This results in considerable nutritional loss and consumer dissatisfaction. Although food and drug packaging are a multi-billion industry, studies on the presence of heavy metal in these packaging materials in India are lacking. The assessment of heavy metals vanadium, cadmium, arsenic and mercury in thirteen types of locally procured food and drug packaging materials was done by qualitative analysis showcasing presence or absence of heavy metals in them and then confirming their presence using quantitative technique of ICP-OES (Inductively coupled plasma optical emission spectrophotometry).

## Methods

This study was conducted at the Department of Biochemistry, Kasturba Medical College, Mangalore, Karnataka and Department of Chemical Engineering, National Institute of Technology, Suratkal, Karnataka. The Institutional Ethics Committee (IEC) approval was obtained prior to the conduct of the study.

A total of eleven food packaging samples and two drug packaging materials were bought from the local market.

The samples included aluminium cans, leak-proof bags, cardboard, tetrapaks, cellophane, tissues, sachets, aluminium bags and boxes, plastic bags and containers, as well as medicinal blister packets and medicinal closures.

The packaging materials were collected, cleaned with distilled water and dried in a hot air oven. The samples were cut into small pieces using scissors and metal cutters, and 10 grams each of the sample were weighed using a calibrated electronic weighing scale. The following tests were performed in triplicates.

### Analysis of toxins in the samples

Packaging material was collected and cleaned as mentioned above. A total of 10 grams of weighed samples were digested using standard acid digestion technique as described by USEPA 305 (United States Environmental Protection Agency amendment no 3050(B). ^20^ Operating conditions for microwave-assisted digestion were followed as per the USEPA 3051. ^21^ The operating conditions were followed as per original protocol. Digestion of the complete sample takes place in the process leaving the heavy metals in solution form. Digested solutions were cooled at room temperature and filtered through 0.45-μm microfilters. Prior to inductively coupled plasma-optical emission spectrometry (ICP-OES) analysis, the samples were again filtered using 0.25-μm filters ensuring a clear filtrate. Analytical grade reagents such as concentrated hydrochloric acid (36%), sulphuric acid (98%) and nitric acid (68%) procured from Sigma Aldrich Chemicals Private Ltd, Bangalore, India were used. Double distilled water was procured from the Chemical Engineering lab at the National Institute of Technology, Karnataka using Accumax Distillation Unit for the extraction of the probable toxins from the samples.

### Qualitative analysis

Post extraction of the sample, preliminary tests were performed to qualitatively confirm the presence of heavy metals.

Spot test for vanadium: The extracted solution was treated with 0.1% sodium salicylate solution in a medium of 20 ml syrupy phosphoric acid. A turquoise blue colour obtained indicated a positive result. ^22^ The blue colour in the test is formed when vanadium forms a complex called vanadium hydroxyamide naphthol ternary complex with the hydrogen-free radical. ^22^

SenSafe Boris’s mercury detection strips were used for the quick and easy detection of low levels of mercury procured by Industrial Test Systems Ltd, U.S.A. Dithizone in the extractant fluid acts as a sensitive reagent for the determination of mercury in acidic media. Dithizone forms coloured primary and secondary dithizonates complexes with mercury. ^23^ The presence of a yellow to ochre colour indicates a positive result for presence of mercury.

Swab test for cadmium: a clean cotton swab was dipped in 70% alcohol and dried, after which it was immersed in the extract and air-dried. The indicator was prepared in a small cup containing 1,5- diphenycarbazone and alcohol (70%) in a concentration of 70:30. The cotton swab dipped in the indicator turns violet-blue colour when confirming the presence of cadmium. The coloured complex is formed when cadmium reacts with diphenycarbazone. ^24^

Aquasol arsenic detector kit was used following the manufacturer’s instructions. Briefly, (i) an arsenic silica reagent (ASR) test paper was placed on the black lid of the test bottle, using a forceps provided and making sure that the hole in the lid was covered by the test paper. (ii) The blue disc on the black lid was fixed gently. (iii) Precaution was taken not to touch the test zone. (iv) A 5 ml sample extract was taken in the test bottle with the syringe provided. (v) Three demitasse spoons of ASR-1 were added and gently swirled for a minute. (vi) Six demitasse spoons of ASR-2 were added to the above and immediately tightly screw-capped with the blue disc as prepared above. (vii) The test bottle was allowed to stand for 15 minutes, with intermittent swirling. (viii) The ASR test paper was removed from the lid and dipped in water for two seconds and excess water shaken off. The colour obtained on the test paper was compared with the colour comparison chart provided, at the end of five and eight minutes. A yellow tint indicated a positive test.

### Quantitative analysis

All thirteen samples were extracted as described above, labelled and subjected to ICP-OES to quantify the chosen heavy metals. Multi-element standard (REICPCAL29A) was used to standardize and calibrate the metal concentration. ^25^ Standards of all four metals were run in triplicates using an inductively coupled optical plasma emission spectrophotometer (ICP-OES) from Agilent Technologies (U.S.A) with Expert software version of 7.100.6821.61355 and firmware version of 2994.

## Results

All tests were run in triplicates to ensure consistency in results. The average of the three readings was taken for computation and analyses.

### Digestion

Microwave-assisted digestion was carried out using hydrochloric acid and sulphuric acid in a concentration of 80:20 ^26^ used for samples like aluminium can and leak-proof bag. Dehydrator aid proof digestion used a combination of sulfuric acid and nitric acid in the ratio of 80:20 was used for medicinal blister packets, tetrapak, sachets, plastic containers and medicinal closures. Acid digestion with a combination of hydrochloric acid and nitric acid in ratio of 80:20 respectively was employed for the digestion of cellophane, tissue cardboard, aluminium bags, box and plastic container. The time taken for digestion ranged from 210 minutes to 34 minutes ( Table 1).

**Table 1.** Extraction of heavy metals from the samples.

Packaging material	Type of extraction	Time taken for extraction (in minutes)
Leak proof bags	Microwave assisted extraction HCl and H _2_SO _4_	210
Plastic container	Dehydrator aid proof extraction H _2_SO _4_	190
Aluminium can	Microwave assisted extraction HCl and H _2_SO _4_	140
Cellophane	Acid digestion 80:20 HCl and HNO _3_	140
Tetrapak	Dehydrator aid proof extraction H _2_SO _4_	130
Sachet	Dehydrator aid proof extraction H _2_SO _4_ and HNO _3_	80
Plastic bag	Acid digestion 80:20 HCl and HNO _3_	60
Cardboard	Acid digestion 80:20 HCl and HNO _3_	60
Medicinal closure	Dehydrator aid proof extraction H _2_SO _4_ and HNO _3_	46
Medicinal blister packet	Dehydrator aid proof extraction H _2_SO _4_ and HNO _3_	46
Aluminium bag	Acid digestion 80:20 HCl and HNO _3_	45
Aluminium box	Acid digestion 80:20 HCl and HNO _3_	42
Tissue	Acid digestion 80:20 HCl and HNO _3_	34

A) Qualitative analysis

Cardboard, medicinal blister packets and closures were positive for the presence for vanadium. Mercury was qualitatively identified in eight samples *viz.* aluminum can and bag, leak-proof bags, sachet, plastic bag, cellophane, medicinal blister packets and closure. Arsenic was present in samples: aluminum bag and box, sachet, cellophane and leak-proof bags. Cadmium was detected in plastic bag, aluminum bag, sachet, cardboard and leakproof bag ( Table 2).

**Table 2.** Presence of heavy metals using qualitative analysis.

Sl No	Packaging material	Vanadium	Mercury	Arsenic	Cadmium
1	Leak proof bags	**-**	**+**	**+**	**+**
2	Plastic container	**-**	**-**	**-**	**-**
3	Aluminium can	**-**	**+**	**-**	**-**
4	Cellophane	**-**	**+**	**+**	**-**
5	Tetrapak	**-**	**-**	**-**	**-**
6	Sachet	**-**	**+**	**+**	**+**
7	Plastic bag	**-**	**+**	**-**	**+**
8	Cardboard	**+**	**-**	**+**	**+**
9	Medicinal closure	**+**	**+**	**-**	**-**
10	Medicinal blister packet	**+**	**+**	**-**	**_**
11	Aluminium bag	**-**	**+**	**+**	**+**
12	Aluminium box	**-**	**-**	**+**	**-**
13	Tissue	**-**	**-**	**-**	**-**

“+” - presence of heavy metal , “-” - absence of heavy metal.

**Figure 1.**
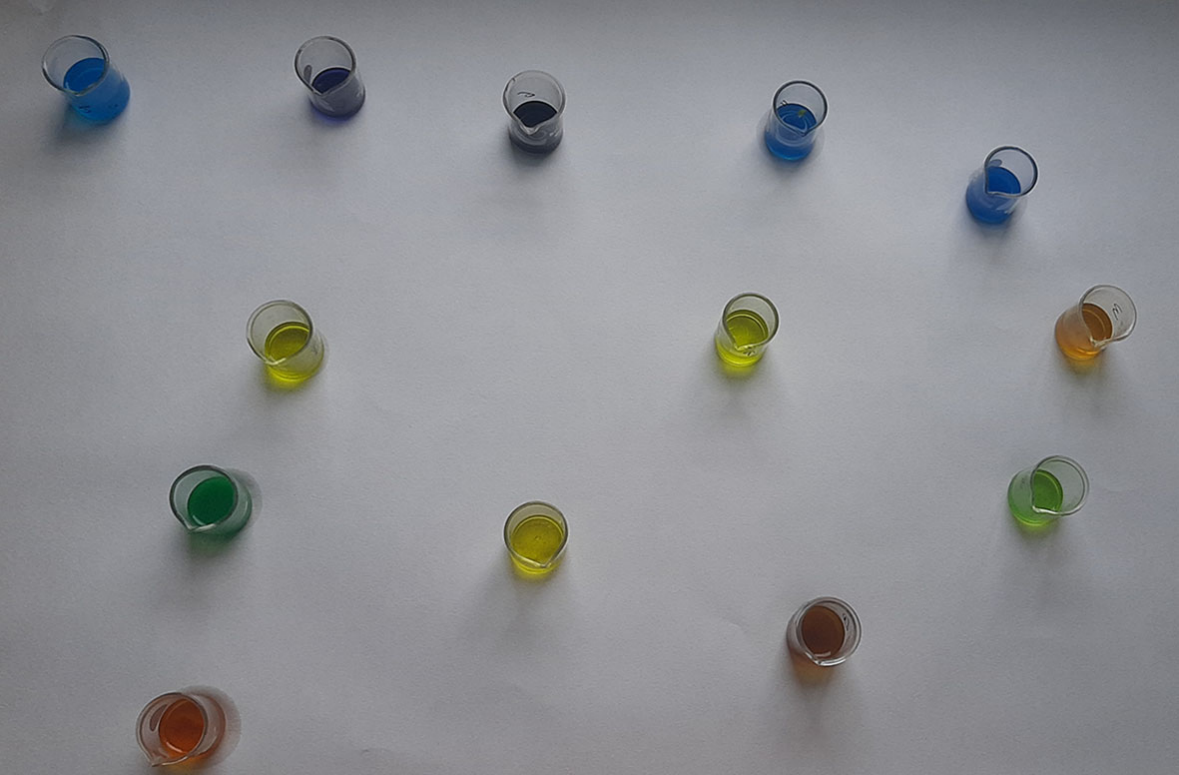
Spot test for vanadium.

**Figure 2.**
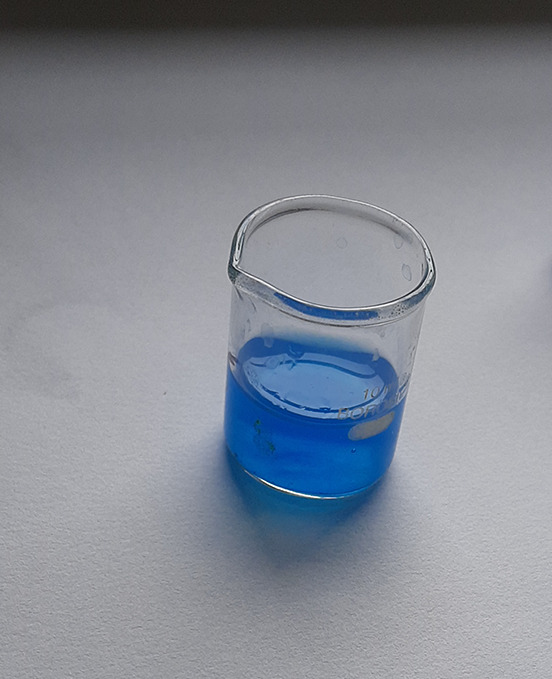
Turquoise colour indicating positive test for vanadium.

**Figure 3.**
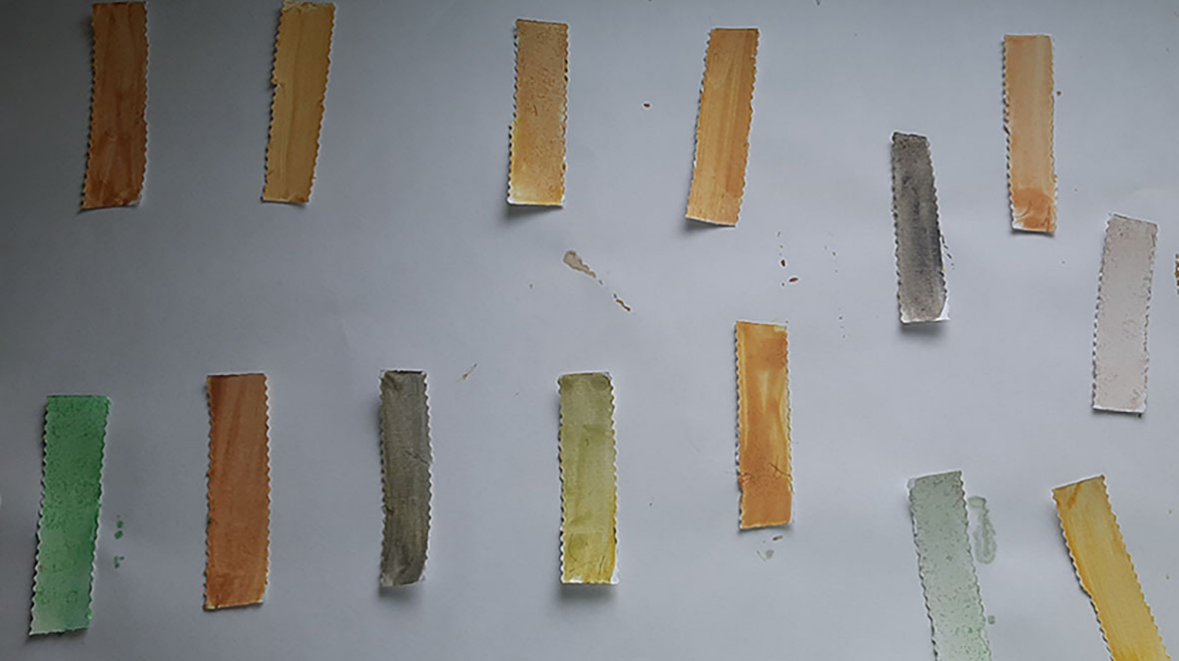
Mercury Boris’s SenSafe strips tested for presence of mercury.

**Figure 4.**
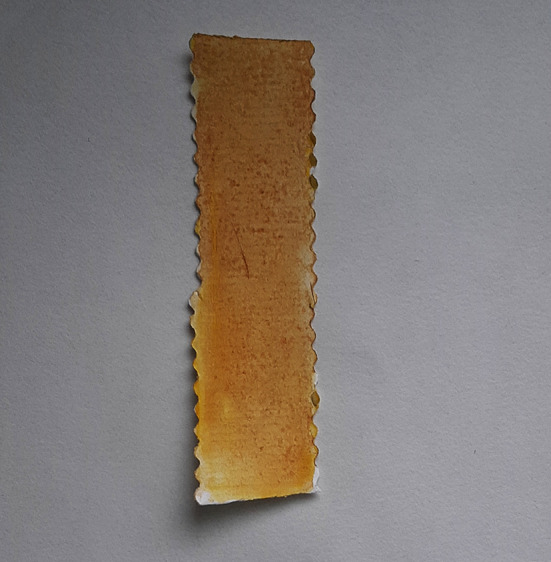
Ochre colour indicates positive test for mercury.

**Figure 5.**
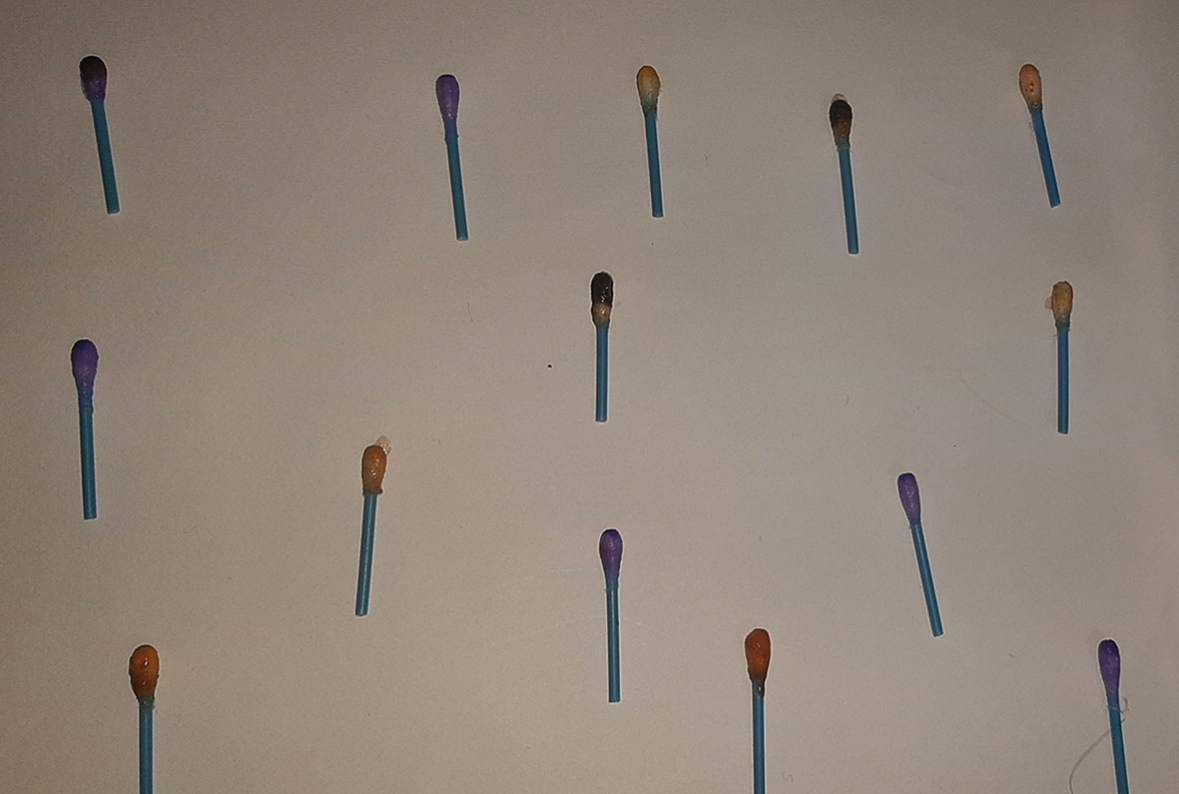
Cotton swab test for cadmium.

**Figure 6.**
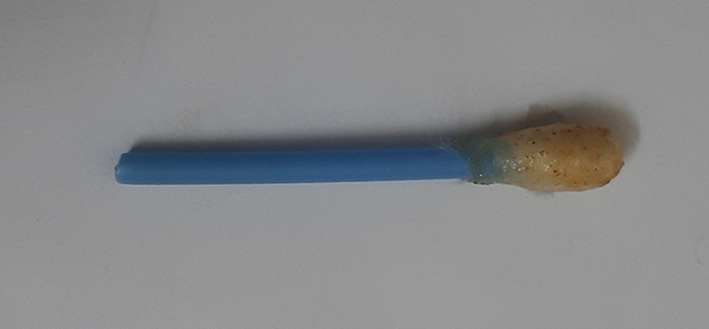
Negative test for cadmium.

**Figure 7.**
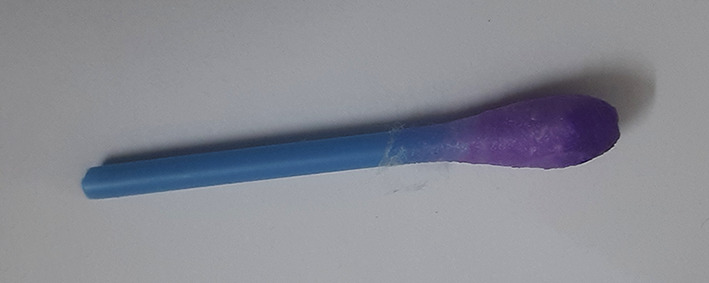
Violet colour indicates positive test for cadmium.

**Figure 8.**
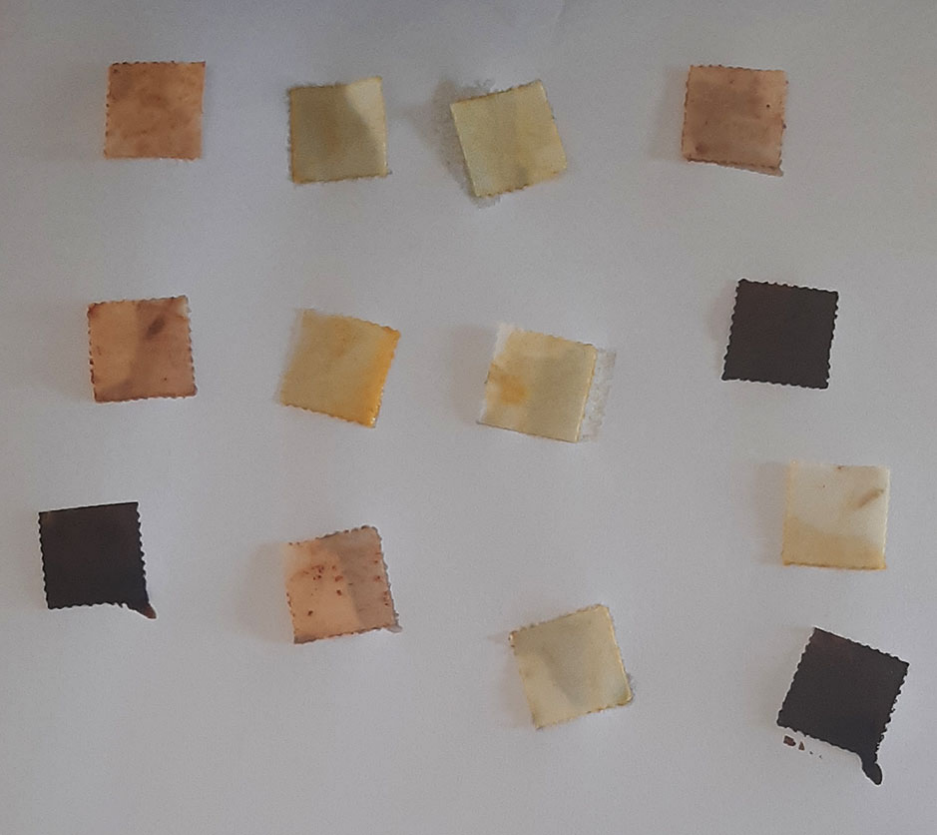
Aquasol arsenic detector test.

**Figure 9.**
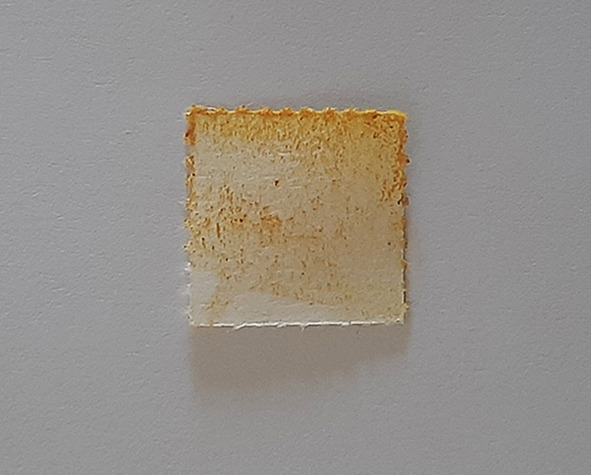
Yellow to ochre indicates positive test for arsenic.

### Quantitative analysis

Heavy metals were detected in the all the thirteen samples. The levels of heavy metals ranged from 0.29 – 40.8 ppm for vanadium,1.7 – 236.2 ppm for arsenic, 1.53 – 546 ppm for mercury and 2.2 – 337 ppm for cadmium. The maximum permitted quantity (ppm) as suggested by the European Council for Food Packaging Association is; As-0.002 ppm, V-0.01 ppm, Hg and Cd being 100 ppm. ^27^ Vanadium was found within the permissible limits in three samples (tissue, sachet, cellophane). Arsenic was above the permissible limit in all the studied samples. Similarly, cadmium was found to be above the acceptable limits only in the sachet. Arsenic was the most common heavy metal contaminant and cadmium was the least ( Table 3).

**Table 3.** Concentrations in parts per million (ppm) of heavy metals detected in study materials. Bold values indicate values of heavy metals in packaging materials below the or under acceptable amount that is within the given range and not higher than the prescribed limit.

Label	Vanadium (V) (ppm)	Arsenic (As) (ppm)	Mercury (Hg) (ppm)	Cadmium (Cd) (ppm)
**Permitted concentration**	**≤0.01**	**≤0.002**	**≤100**	**≤100**
**Packaging material**				
Aluminium can	0.59	3.3	**3.2**	**ND**
Leak proof bags	0.49	86	**5.6**	**3.50**
Cardboard	40.8	0.83	**17.5**	**35.6**
Tetrapak	0.92	1.5	**9.75**	**10.0**
Cellophane	**ND**	2.61	**2.03**	**0.65**
Tissue	**0.097**	94.3	**1.72**	**1.5**
Sachet	**ND**	1.7	546	337
Aluminium bag	0.29	29.5	**3.00**	**16.6**
Aluminium box	0.84	123	**1.53**	**2.20**
Plastic bag	35.6	62.2	174	**34.4**
Plastic container	0.88	2.80	**9.10**	**ND**
Medicinal closure	19.6	236.2	**19.6**	**0.28**
Medicinal blister packet	0.98	0.54	**4.50**	**ND**

ND = not detected.

**Table 4.** Nomenclature.

Symbol	Meaning
V	Vanadium
As	Arsenic
Hg	Mercury
Cd	Cadmium
ppm	Parts per million
ml	Milliliter
%	Percentage
ICP-OES	Inductively coupled plasma optical emission spectrophotometry
USEPA	United States Environmental Protection Agency
ASR	Arsenic silica reagent
IARC	International Agency for Research on Cancer
WHO	World Health Organization
NTP	The National Toxicology Programme
NIH	National Institute of Health
FDA	Food and drug administration
CDC	Center for Disease Control and Prevention

## Discussion

In the current study, eleven commercially available food packaging materials and two drug packages were analysed for the presence of four heavy metals, namely vanadium, arsenic, mercury and cadmium. Packaging materials constitute the mainstay of the modern food industry. Hence, it is important to study their interference with food/drug present within the packaging material. As research has shown leaching of toxins from packaging materials, monitoring the heavy metal leaching is essential to prevent harmful effects to the human body. ^6^

Vanadium is a heavy metal which is used as an additive in stainless steel as well as a catalyst for manufacturing sulfuric acid. It is also used in glass coating and lacquering in aluminum cans to give strength to the material. Vanadium is used as an amalgam in manufacturing cans with stainless steel to give it tensile strength. Vanadium toxicity is known to cause abdominal discomfort by interfering with mucosal lining leading to nausea, bloating, diarrhea and vomiting in the initial stages. Prolonged exposure can result in renal and neural damage. ^28^ Qualitative tests detected the presence of vanadium in only three samples. However, ICP-OES values showed presence of vanadium higher than permissible amounts in 10 samples. The study by Imtiaz *et al.* ^29^ stated that vanadium causes oxidative stress in human cells and alteration in human metabolism, reduces enzymatic activities and disturbs membrane integrity in humans. ^29^ This oxidative stress leads to the formation of pentavalate vanadium which is its most toxic and mobile form.

Arsenic, found in higher concentration in all the study materials (detected by six samples qualitatively), is a heavy metal that can act as a toxin due to its high presence in water which is used for material cleaning and lacquering processes. ^30^ Arsenic is also known to be present as one of the food packaging colorants used in the packaging industry. ^31^ Arsenic could enter the food packaging material during the cleaning process or as an adulterant of iron source. Arsenic has been used as a common ingredient in many pesticides and herbicides in the past. It is known to cause skin lesions and cancer in humans. ^32^ The International Agency for Research on Cancer (IARC), a part of the World Health Organisation (WHO) has stated that exposure to arsenic is the leading cause of lung, bladder and skin cancer. ^33^ The National Toxicology Programme (NTP) is composed of several different government agencies, including the National Institute of Health (NIH), the Center for Disease Control and Prevention (CDC), and the Food and Drug Administration (FDA). In its most recent *Report on Carcinogens,* the NTP classifies arsenic and inorganic arsenic compounds as known to be human carcinogens. The study by Park *et al.* stated that arsenic is present in paper in contact with food such as any kind of wrapping, and paper board, and presents a risk to consumer safety as increased ingestion in humans can cause skin lesions and gastric cancers. ^34^

Mercury dissolves in aluminum at room temperature and is added as an additive in aluminum cans and foils to enhance the shelf-life of the seafood packed inside. ^35^ Ingestion of mercury is the leading cause of Minamata disease. Mercury was detected qualitatively in eight samples and quantitatively in amounts greater than those permissible in sachet and plastic bag only. The mercury absorbed in the body mainly concentrates in the kidneys and brain. ^36^ The half-life of mercury in the body is about 70 days. Inorganic mercury is mainly absorbed through the respiratory tract, but is also absorbed through the skin to a small extent (3-4%) or gastrointestinal (GI) tract (2–10%). Methylmercury is easily absorbed into the GI tract (≥95%) and into the respiratory tract (≈80%). About 90% of methylmercury is excreted through the faeces via bile, and less than 10 % through urine. The absorbed mercury is distributed throughout all tissues within 30 hours. Its half-life ranges from 45 to 70 days. ^37^ Higher concentrations (more than 100 ppm) of mercury have been known to cross the placenta and result in foetal defects. ^14^ Toxic effects of mercury have been reported even at lower concentrations in humans. ^38^^–^^40^ Therefore, it would be worthwhile to study the leaching from the packaging material and reconsider the admissible limit if proven.

Ingestion of cadmium in higher concentration creates oxidative stress in the cells and increases the level of antioxidant uptake to protect against macromolecular cell damage, thus leading to prolonged exposure to cadmium, causing depletion of antioxidant levels in the body. Cadmium is generally a contaminant present as residues of the recycling and manufacturing processes, and hence determining their suitability in packaging materials coming in direct contact with foodstuffs is imperative. During the recycling process and cooling of cans, cadmium enters the food production system due to changes in temperature or as an additive used in the metal and glass industry. Cadmium can cause respiratory illnesses, lung fibrosis and cancer. ^41^ Huff *et al.*, found cadmium to be the major causative agent of lung cancer, and possibly prostate cancer. Studies in experimental animals have demonstrated that cadmium concentrations higher than those set by regulations (100 ppm) cause tumours at multiple tissue sites, by various routes of exposure, and in several species and strains. ^42^

It is alarming to find high concentrations of toxic metals in several of the packaging materials studied. While cadmium seemed to be the least abundant contaminant, arsenic was the most prevalent heavy metal contaminant. Of all the types of packaging materials studied, sachets, which are manufactured using a plastic and aluminium amalgam, had the highest concentration of all the heavy metals.

## Conclusions

In summary, a wide variety of food and drug packaging materials from an Indian market were tested for the presence of heavy metal, namely vanadium, arsenic, cadmium and mercury. Our study shows the presence of heavy metals in routinely used food packaging materials, measured quantitatively and qualitatively. All samples, irrespective of the results of qualitative analysis, were subjected to quantitative analysis, as ICP-OES is a sensitive technique for the detection of heavy metals. Of the thirteen samples analysed, arsenic was found in all samples at concentrations above the regulated limits; cadmium was present in higher than regulated concentrations only in sachets. Arsenic was present in all samples, whereas vanadium was present in lower concentration in tissue, cellophane and sachets; mercury was present in higher concentration in sachets and plastic bags. Leaching of these heavy metals into the packaged food/drug may be potential health hazard that severely compromise the well-being of humans and the environment. This study calls for stringent regulatory guidelines and strict monitoring of packaging materials at all stages, starting from raw material selection, storage and production until it reaches the consumer. The post-packaging handling, transport, storage and conservation of the supply chain requirements further add to prospective leaching, which may be the scope of future studies.

## Data availability

### Underlying data

Mendeley data: ICP-OES data for “Assessment of toxins in food and drug packaging materials”, https://data.mendeley.com/datasets/gnwy7nzpnt/1. ^43^

This project contains the following underlying data:

- SENNA As KMC 6.12.21.docx (Arsenic ICP-OES data)
- SENNA Hg KMC 6.12.21.docx (Mercury ICP-OES data)
- SENNA KMC Cd 6.12.21.docx (Cadmium ICP-OES data)
- SENNA kmc V 6.12.21.docx (Vanadium ICP-OES data)
- Table 1. docx

Data are available under the terms of the Creative Commons Attribution 4.0 International license (CC-BY 4.0).
