# Supplementary material for: Assessment of Arsenic, Vanadium, Mercury, and Cadmium  in Food and Drug Packaging
Source: F1000Res. 2024 Mar 4;11:648. Originally published 2022 Jun 13. [Version 3] doi: 10.12688/f1000research.121473.3 (PMC11109717; doi:10.12688/f1000research.121473.3)
Supplement: Peer review history

# Reviewer report: Reviewer response for version 3

**DOI:** 10.5256/f1000research.163010.r252513

**Published:** 2024-03-18

**Authors:** Marin Senila

**Affiliations:** 1 National Institute for Research and Development of Optoelectronics INOE 2000 INCD Bucharest, Research Institute for Analytical Instrumentation, Cluj-Napoca, Romania

**Editorial note (15th May 2024): the reviewer's report for Version 3 of this article was originally published on 18th March 2024, but the reviewer had not yet been able to view the authors' direct response to their previous comments due to a delay with processing this response from the editorial side. The reviewer has since been notified of the authors' direct response and after reading this response the reviewer has now updated their report status and comments to "Approved".**

 Approved.

Is the work clearly and accurately presented and does it cite the current literature?

Partly

If applicable, is the statistical analysis and its interpretation appropriate?

No

Are all the source data underlying the results available to ensure full reproducibility?

Partly

Is the study design appropriate and is the work technically sound?

No

Are the conclusions drawn adequately supported by the results?

Partly

Are sufficient details of methods and analysis provided to allow replication by others?

No

Reviewer Expertise:

Atomic spectrometry, metals determination, ICP-OES

I confirm that I have read this submission and believe that I have an appropriate level of expertise to confirm that it is of an acceptable scientific standard.

# Reviewer report: Reviewer response for version 2

**DOI:** 10.5256/f1000research.152481.r229064

**Published:** 2024-02-19

**Authors:** Marin Senila

**Affiliations:** 1 National Institute for Research and Development of Optoelectronics INOE 2000 INCD Bucharest, Research Institute for Analytical Instrumentation, Cluj-Napoca, Romania

In the manuscript entitled “Assessment of heavy metals in food and drug packaging materials”, authors tried to assess the concentration of some heavy metals in several food and drug packaging materials. There are many necessary improvements, the most important:

1. The title is too general and should be rewritten to be more specific for the conducted research. 
2. The correct name of the ICP-OES technique is inductively coupled plasma-optical emission spectrometry (as it is written in the Abstract), not Inductively coupled plasma optical emission spectrophotometry (as it is written in the Introduction). It is not necessary to give the principle of functioning of this technique; it is well-known. 
3. Arsenic is a metalloid; it is not a metal; please mention that.
4. No quality control is provided for the digestion method and for instrumental determination. How do you check that the results are correct?
5. The authors state that the ICP-OES can detect heavy metals from 0.0002 ppm to 1000 ppm range. It depends on the metal. For mercury, a studied analyte in this research, the range of concentrations is much limited. The authors should provide the range of calibration curves used in their own study, working ranges for each analyte, limits of detection and limits of quantification. For quantitative analysis, the LOQ is necessary; the authors reported in Table 3 ND as not detected, but below LOQ should be given.
6. For Table 3, the cited reference for the permitted concentration of metals in packaging materials should be provided. Strangely, the permitted concentration of Hg and Cd (known as very toxic elements) is 50000 times higher than for As, and 10000 times higher than for V. Please check the reference and measurement units.
7. Generally, the Discussion is too general and contains well-known literature information about metal toxicity. Please discuss your results in relation to measured concentration and maximum admitted values and compare your data with previous reports.

Is the work clearly and accurately presented and does it cite the current literature?

Partly

If applicable, is the statistical analysis and its interpretation appropriate?

No

Are all the source data underlying the results available to ensure full reproducibility?

Partly

Is the study design appropriate and is the work technically sound?

No

Are the conclusions drawn adequately supported by the results?

Partly

Are sufficient details of methods and analysis provided to allow replication by others?

No

Reviewer Expertise:

Atomic spectrometry, metals determination, ICP-OES

I confirm that I have read this submission and believe that I have an appropriate level of expertise to state that I do not consider it to be of an acceptable scientific standard, for reasons outlined above.

# Response

**Published:** 2024-02-21

**Authors:** SENNA Mukhi

**Affiliations:** Kasturba Medical College, Mangalore, Manipal Academy of Higher Education (MAHE), India

**Query 1 -** The title is too general and should be rewritten to be more specific for the conducted research. 

**Response 1 – **The title is  changed to – Assessment of Arsenic, Vanadium, Mercury, and Cadmium in Food and Drug Packaging

**Query 2** - The correct name of the ICP-OES technique is inductively coupled plasma-optical emission spectrometry (as it is written in the Abstract), not Inductively coupled plasma optical emission spectrophotometry (as it is written in the Introduction). It is not necessary to give the principle of functioning of this technique; it is well-known. 

**Response 2** - The changes are made in the manuscript. 

**Query 3** - Arsenic is a metalloid; it is not a metal; please mention that.

**Response 3**- Arsenic is classified chemically as a metalloid, having both properties of a metal and a nonmetal; however, it is frequently referred to as a metal. It is referred  to as a metal at ATSD website, and therefore we have  mentioned the same in the entire manuscript

 Reference – Agency of toxic substances and diseases. https://www.atsdr.cdc.gov/

**Query 4** - No quality control is provided for the digestion method and for instrumental determination. How do you check that the results are correct?

**Response 4** – At first, calibration of each heavy metal is conducted only then samples are run in the machine for detection of heavy metal. Calibration curves in Inductively Coupled Plasma Optical Emission Spectrometry (ICP-OES) are crucial for accurately quantifying element concentrations in samples. They establish a relationship between signal intensity and concentration, ensuring precise measurements in unknown samples. Calibration curves also demonstrate the instrument's linear response to varying concentrations, ensuring accurate measurements across a wide range. They also serve as quality control measures, verifying instrument performance and providing a standardized method for consistent results across different analyses and laboratories. Calibration curves (Figure 10) enclosed in the manuscript.

**Query 5** - The authors state that the ICP-OES can detect heavy metals from 0.0002 ppm to 1000 ppm range. It depends on the metal. For mercury, a studied analyte in this research, the range of concentrations is much limited. The authors should provide the range of calibration curves used in their own study, working ranges for each analyte, limits of detection and limits of quantification. For quantitative analysis, the LOQ is necessary; the authors reported in Table 3 ND as not detected, but below LOQ should be given.

**Response 5** - The detection limits are stated in the standard curves provided viz., V- 0.000011ppm, As- 0.000156ppm, Cd- 0.000442 ppm, Hg- <0.001.

**Query 6** - For Table 3, the cited reference for the permitted concentration of metals in packaging materials should be provided. Strangely, the permitted concentration of Hg and Cd (known as very toxic elements) is 50000 times higher than for As, and 10000 times higher than for V. Please check the reference and measurement units.

**Response 6** -   The maximum permitted quantity (ppm) as suggested by the European Council for Food Packaging Association (Nordic guidance for authorities) is; As-0.002 ppm, V-0.01 ppm, Hg – 0.003 ppm, Cd, 0.005 ppm.  This change has been incorporated. The updated reference no is 27 in the manuscript and is also stated below.

 Reference - Cederberg, D.L., Christiansen, M., Ekroth, S., Engman, J., Fabech, B., Guðjónsdóttir, K., Mikkelsen, B., et al. (2015) Food Contact Materials—Metals and Alloys. Vol. 2015522, Nordic Guidance for Authorities, Industry and Trade, Nordic Council of Ministers. https://doi.org/10.6027/TN2015-522

**Query 7** - Generally, the Discussion is too general and contains well-known literature information about metal toxicity. Please discuss your results in relation to measured concentration and maximum admitted values and compare your data with previous reports.

**Response 7 - **The discussion section has been elaborated in relation to measured concentration and maximum admitted values and in comparison, with previous reports. The additional references (Ref 43 – 48) have been incorporated.****

# Reviewer report: Reviewer response for version 2

**DOI:** 10.5256/f1000research.152481.r185018

**Published:** 2023-07-24

**Authors:** Mahesh Ganesapillai

**Affiliations:** 1 School of Chemical Engineering, Vellore Institute of Technology, Vellore, India

The queries are addressed. The article can be accepted for indexing.

Is the work clearly and accurately presented and does it cite the current literature?

Partly

If applicable, is the statistical analysis and its interpretation appropriate?

Not applicable

Are all the source data underlying the results available to ensure full reproducibility?

Partly

Is the study design appropriate and is the work technically sound?

Yes

Are the conclusions drawn adequately supported by the results?

Yes

Are sufficient details of methods and analysis provided to allow replication by others?

Partly

Reviewer Expertise:

Solid Waste Management; Closed loop, Nutrient recovery, Separation Technology

I confirm that I have read this submission and believe that I have an appropriate level of expertise to confirm that it is of an acceptable scientific standard.

# Reviewer report: Reviewer response for version 1

**DOI:** 10.5256/f1000research.133336.r178554

**Published:** 2023-06-29

**Authors:** Xin-Yuan Huang

**Affiliations:** 1 Nanjing Agricultural University, Nanjing, China

In this manuscript, authors quantified four heavy metals in 13 types of food and drug packaging materials, including arsenic (As), vanadium (V), mercury (Hg) and cadmium (Cd). They found that  10 of 13 samples are above the permissible range for vanadium, all samples for arsenic, two samples for mercury and one sample for cadmium. This manuscript was well written but I have the following concerns.

1. Concentrations of the 4 heavy metals shown in Table 3 are presented as parts per million (ppm), which represent the concentrations of these 4 heavy metals in the extract buffer (probably after dilution but not mentioned in the methods). The concentrations of heavy metals in the extract buffer depends on the amount of the food and drug packaging materials used for extraction. So the concentrations must be normalized to the weight of the materials.
2. The same extraction method was used for all eleven food packaging samples and two drug packaging materials. How to make sure they have the same extraction efficiency?
3. Quantitative analysis of 4 heavy metals were determined by ICP-OES. What are detection limits for arsenic (As), vanadium (V), mercury (Hg) and cadmium (Cd) in this instrument?
4. Blank control samples must be included when run samples on ICP-OES. Without blank controls, it is hard to rule out the presence of heavy metal coming from the samples or simply from reagents for extraction or the comtamination during the extration. 

Is the work clearly and accurately presented and does it cite the current literature?

Partly

If applicable, is the statistical analysis and its interpretation appropriate?

Not applicable

Are all the source data underlying the results available to ensure full reproducibility?

Partly

Is the study design appropriate and is the work technically sound?

No

Are the conclusions drawn adequately supported by the results?

Partly

Are sufficient details of methods and analysis provided to allow replication by others?

Yes

Reviewer Expertise:

Plant ionomics

I confirm that I have read this submission and believe that I have an appropriate level of expertise to state that I do not consider it to be of an acceptable scientific standard, for reasons outlined above.

# Response

**Published:** 2023-07-01

**Authors:** Rukmini M S

**Affiliations:** Kasturba Medical College, Mangalore, Manipal Academy of Higher Education,Manipal, India

1. As per standard deduction method it represents the digestion of each packaging material having concentration of vanadium, arsenic, mercury and cadmium, when 10 gram of sample was digested. Accordingly, the experiment is performed and analysed and the concentration of heavy metal is determined in ppm.

 2. The word used in place of extraction is digestion, the same correction would be made in the updated version of the manuscript. Digestion of complete sample takes places in the process leaving the heavy metal in solution form and therefore the accuracy was 100%.

 3. The detection for arsenic (As), vanadium (V), mercury (Hg) and cadmium (Cd) for our study was carried out in ppm (parts per million). The machine is highly sensitive and detects these heavy metals in ppm (parts per million), ppb (parts per billion )  and ppq (parts per quadrillion).  For heavy metal samples ICP -OES can detect heavy metals from 0.0002ppm to 1000ppm range. This information would be added in the updated version of the manuscript.

 ​​​​​​​4. The analysis was done with standards of heavy metals namely vanadium, cadmium, mercury and arsenic. For all these heavy metals, calibration curve was developed using standard solution and then the concentration was determined. Thus, no control was required as same solution was used for digestion.

# Reviewer report: Reviewer response for version 1

**DOI:** 10.5256/f1000research.133336.r154512

**Published:** 2022-11-11

**Authors:** Mahesh Ganesapillai

**Affiliations:** 1 School of Chemical Engineering, Vellore Institute of Technology, Vellore, India

The study conducted is well aligned with the scope of the journal and boasts considerable novelty in its approach. However, there are a few possible revisions which may alleviate the overall quality of the work:

1. The objective of the study needs to be clearly outlined in the last paragraph of introduction. Please refer the following articles for a proper understanding of the expected phrasing ^1^^,^^2^^,^^3^.
2. **Introduction**: Information on the various quantitative analyses conducted must be mentioned in this section. Such details provide readers with valuable context for the study and improves the logical flow of the manuscript.
3. **Extraction**: “…a concentration of 80:20 used…” Why was this composition chosen? Please provide valid reference for the same.
4. **Table 2**: The data represented lacks clarity. Consider incorporating a legend representing the symbols.
5. There are several abbreviations in the manuscript – please include a nomenclature table for the convenience of readers.
6. **Conclusions**: The section needs further improvement and inclusion of better inferences. The mere mention of the presence of heavy mental entities does not conclude the study adequately.

Is the work clearly and accurately presented and does it cite the current literature?

Partly

If applicable, is the statistical analysis and its interpretation appropriate?

Not applicable

Are all the source data underlying the results available to ensure full reproducibility?

Partly

Is the study design appropriate and is the work technically sound?

Yes

Are the conclusions drawn adequately supported by the results?

Yes

Are sufficient details of methods and analysis provided to allow replication by others?

Partly

Reviewer Expertise:

Solid Waste Management; Closed loop, Nutrient recovery, Separation Technology

I confirm that I have read this submission and believe that I have an appropriate level of expertise to confirm that it is of an acceptable scientific standard, however I have significant reservations, as outlined above.

## References

[1] : Design and Analysis of Artificial Neural Network (ANN) Models for Achieving Self-Sustainability in Sanitation. *Applied Sciences* .2022;12(7) : 10.3390/app12073384 10.3390/app12073384

[2] : The face behind the Covid-19 mask - A comprehensive review. *Environ Technol Innov* .2022;28: 10.1016/j.eti.2022.102837 102837 10.1016/j.eti.2022.102837 35879973 PMC9299984

[3] : Regular antenatal care visits were associated with low risk of low birth weight among newborns in Rwanda: Evidence from the 2014/2015 Rwanda Demographic Health Survey (RDHS) Data. *F1000Research* .2022;10: 10.12688/f1000research.51969.2 10.12688/f1000research.51969.2 PMC964110736406958

# Response

**Published:** 2022-11-18

**Authors:** Rukmini M S

**Affiliations:** Kasturba Medical College, Mangalore, Manipal Academy of Higher Education,Manipal, India

1. The objective of the study will be clearly stated and suggestions will be incorporated.

 2. The changes suggested in the introduction will be made.

 3. Regarding the extraction procedure: 'The Aqua Regia is a standard acid mixture of HCl:HNO3 ( *volume* ratio is usually 4 parts concentrated hydrochloric acid to 1 part concentrated nitric acid) prepared to digest most of the samples for the metal analysis.' 

 4. **Table 2**: The legends have been inadvertently omitted while editing. We will make the changes as per the suggestion.

 5. We will include a nomenclature table for abbreviations or incorporate the complete metal name, wherever applicable.
